# Quality of life and the risk of contracting malaria by multivariate analysis in the Brazilian Amazon region

**DOI:** 10.1186/1475-2875-13-86

**Published:** 2014-03-10

**Authors:** Sergio GL Junior, Vanessa MS Pamplona, Tereza CO Corvelo, Edson MLS Ramos

**Affiliations:** 1 Instituto de Ciências Exatas e Naturais, Universidade Federal do Pará, Rua Augusto Corrêa, N. 01, Guamá, CEP 66075-110 Belém, Pará, Brazil; 2 Universidade Federal Rural da Amazônia, Rodovia PA 256, Km 6, S/N, Nova Conquista, CEP 68625-970 Paragominas, Pará, Brazil; 3 Instituto de Ciências Biológicas, Universidade Federal do Pará, Rua Augusto Corrêa, N. 01, Guamá, CEP 66075-110 Belém, Pará, Brazil

**Keywords:** Risk factors, Socioeconomic factors, Epidemiology, Brazilian Amazon, *Plasmodium*

## Abstract

**Background:**

The incidence of malaria in the Amazon basin is closely related to social inequalities, given that precarious economic and socio-environmental conditions represent favourable factors for the transmission of the disease in tropical regions, such as the Brazilian state of Pará. In the present study, an association was found between the variation in a quality of life index (QLI), based on the socioeconomic differences between the municipalities of this state, and the risk of contracting malaria, based on the Annual Parasitic Index (API), with the primary objective of providing guidelines for the development of effective strategies for the control of the disease.

**Methods:**

The API scores for the years between 2003 and 2011 were collected from the Brazilian Ministry of Health’s DATASUS database, and socioeconomic data for the 143 municipalities of Pará were obtained from the 2010 census. The data were analysed using multivariate factorial and correspondence techniques.

**Results:**

The QLI was calculated for each municipality, of which, 69.23% were classified as having a poor or regular quality of life. The municipalities with poor QLI scores also presented moderate to high rates of malaria, with probabilities of 80.97% and 95.13%, respectively, while those with good QLI scores had low rates of malaria, with a probability of 79.24%. The results indicated a concentration of malaria in the south-west of the state of Pará, with an increase of 8.82% in the incidence of the disease over the study period, and the northeastern and Marajó mesoregions, where there was an increase of over 90%. In south-eastern Pará, by contrast, there was a marked reduction (78%) in the incidence of the disease, reflecting the heterogeneous distribution of malaria among the different municipalities and mesoregions of the state, especially those with moderate to high risk of transmission.

**Conclusions:**

These findings confirm that malaria is endemic to Pará and is typical of the state’s poorest areas, and that the distribution of the disease within the state indicates an intimate relationship with the living conditions of the population, affecting primarily the economically less privileged sectors of the society.

## Background

Malaria is an infectious disease found in more than 100 countries in Central and South America, Africa, and Asia. Approximately 40% of the world’s population has contracted the disease at some time. An ever-growing number of people are becoming infected with malaria, principally in tropical regions, such as the Amazon basin, in which the Brazilian state of Pará is located. Most of the Brazilian Amazon basin is characterized by environmental conditions that are favourable to the proliferation of the mosquito vector, and thus the transmission of malaria. A range of ecological, environmental, and socio-economic conditions also influence the living conditions and health of local populations within the state of Pará. The combination of these factors in the region reinforces the need for more effective government initiatives for the control of the disease, and in particular to impede its proliferation.

The incidence of malaria tends to be closely related to social inequalities, given its prevalence in developing regions, in particular poor rural populations, where it has an enormous socio-economic impact [1,2]. In the 1960s and 1970s, Brazil underwent a rural “exodus”, when a large portion of the rural population moved to urban centres, with an accompanying shift in the distribution of endemic diseases [3]. This process resulted in the expansion of peripheral neighbourhoods, which hampered the control of endemic diseases, and demanded new public health strategies from urban administrators.

The present study evaluates the quality of life of the inhabitants of the municipalities of the Brazilian state of Pará with the aim of identifying their socio-economic differences, and possible associations between the risk of contracting malaria, based on the annual parasitic index, and the quality of life of the local population. The results of the analysis provide important guidelines for the development of effective public health measures for the control of malaria in Pará.

## Methods

### Study area

The state of Pará, the capital of which is the city of Belém, is located in northern Brazil, in the Amazon region. The state covers a total area of 1,247,955 km^2^, divided in 143 municipalities, with a total population of 7,581,051 inhabitants [4], and a density of 6.07 inhabitants per square kilometre. The climate of Pará is equatorial, and is characterized by relatively high and constant temperatures and high humidity. Mean annual temperatures range between 24°C and 26°C, while annual precipitation may be as much as 2,900 mm [5].

**Figure 1:**
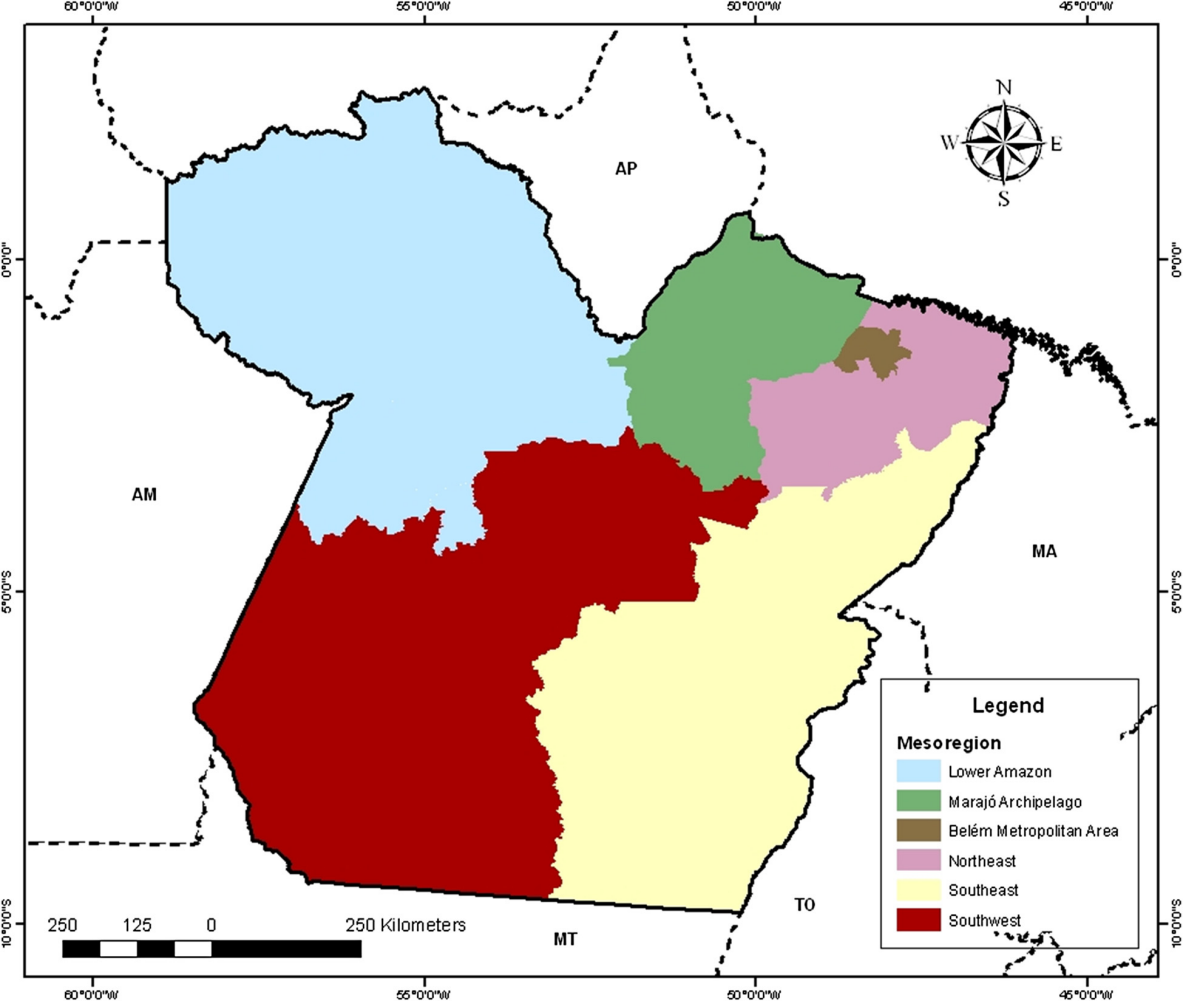
Division of Pará’s cities in Six Mesoregions.

To better visualize the incidence of malaria in the state of Pará, was considered the division of municipalities in six mesoregion: Lower Amazon, Belém Metropolitan Area, Marajó Archipelago, Northeast, Southeast and Southwest (Figure 1). This division was created with the purpose of better monitoring of public policies and applications, and supports the planning, studies and identification of spatial structures of metropolitan regions and other forms of urban and rural agglomerations [4].

### Data collection

Data on the incidence of malaria in the state of Pará between 2003 and 2011 were obtained from the Secretary of Sanitary Vigilance of the Brazilian Health Ministry, through its National Health Information System (DATASUS). Information was obtained on the Annual Parasite Index (API), and the total numbers of reported cases attributed to the three *Plasmodium* species – *Plasmodium falciparum*, *Plasmodium vivax* and *Plasmodium malariae.*

The API is the number of positive tests for malaria per thousand inhabitants in a given region during the year under consideration. Based on their APIs, the municipalities were classified according to the degree of risk of contracting malaria, with values of over 50 being considered high risk, values of 10–49.9, moderate risk, and scores of less than 9.9 being classified as low risk.

### Selection of socio-economic variables

Socio-economic data were obtained from the Brazilian Institute for Geography and Statistics, [4], for the 143 municipalities that make up the state of Pará. Information was collated on (i) per capita income – the mean declared monthly income in multiples of the minimum wage (ii) literacy rate – the percentage of the population able to read and write, (iii) basic sanitation – percentage of households connected to public sewage or drainage system, (iv) refuse collection – percentage of households with public refuse collection, and (v) public water supply – percentage of households connected to a public water supply.

### Statistical analysis

Factorial analysis is a multivariate analysis of interdependence that reduces the data to indices that represent the original variables [6]. Initially, the normality of the data was verified, in order to confirm their suitability for the analysis, followed by the analysis of a correlation matrix, which revealed a substantial number of correlations higher than 0.30. The Kaiser-Meyer-Olkin (KMO) measure of sampling accuracy was also calculated. This index varies from zero to one, with values closer to one being more appropriate for the factorial analysis. Similarly, Bartlett’s test of sphericity was used to evaluate the hypothesis that the correlation matrix is the identity matrix, and finally, the anti-image matrix was analysed, to produce a Measure of Sample Adequacy (MSA), with values closest to one being the most adequate for factorial analysis.

The factorial analysis was applied to the data from 2011, based on a principal components analysis (PCA), for the definition of the two main factors, which were rotated orthogonally by the Varimax method. Factorial scores were calculated by multiplying each of the original data points by their respective factorial weights. A Quality of Life Index (QLI) for each municipality was derived from the sum of factors 1 and 2.

Higher QLI scores indicate a better quality of life in the respective municipality, based on the parameters analysed. The municipalities were classified by percentile, with those presenting factorial scores below P_35_ being classified as poor, values of P_35_ to P_69_ as regular, and those above P_70_ as good, that is, the best quality of life. The factorial analysis was run in SPSS, version 17.0.

The classification of the municipalities according to the quality of life of their populations was analysed using a correspondence analysis - CA [6]. The first step was to analyse the potential association between the quality of life (QLI) and the risk of contracting malaria, based on the API for 2011. The β criterion was then calculated in order to verify the degree of association between the categories of these variables. The results of this analysis supported the use of the CA. The standardized residuals of this analysis permitted the calculation of the probability of occurrence of the value observed in the contingency table, that is, the probability of occurrence of the associations identified by the *χ*^2^ test and β criterion. The CA was run in STATISTICA, version 6.0. A 5% significance level (α) was adopted for all analyses.

### Ethical considerations

The databases are secondary informations from the sources of public access. Consequently this study protocol was realized without Ethical approval.

## Results

**Figure 2:**
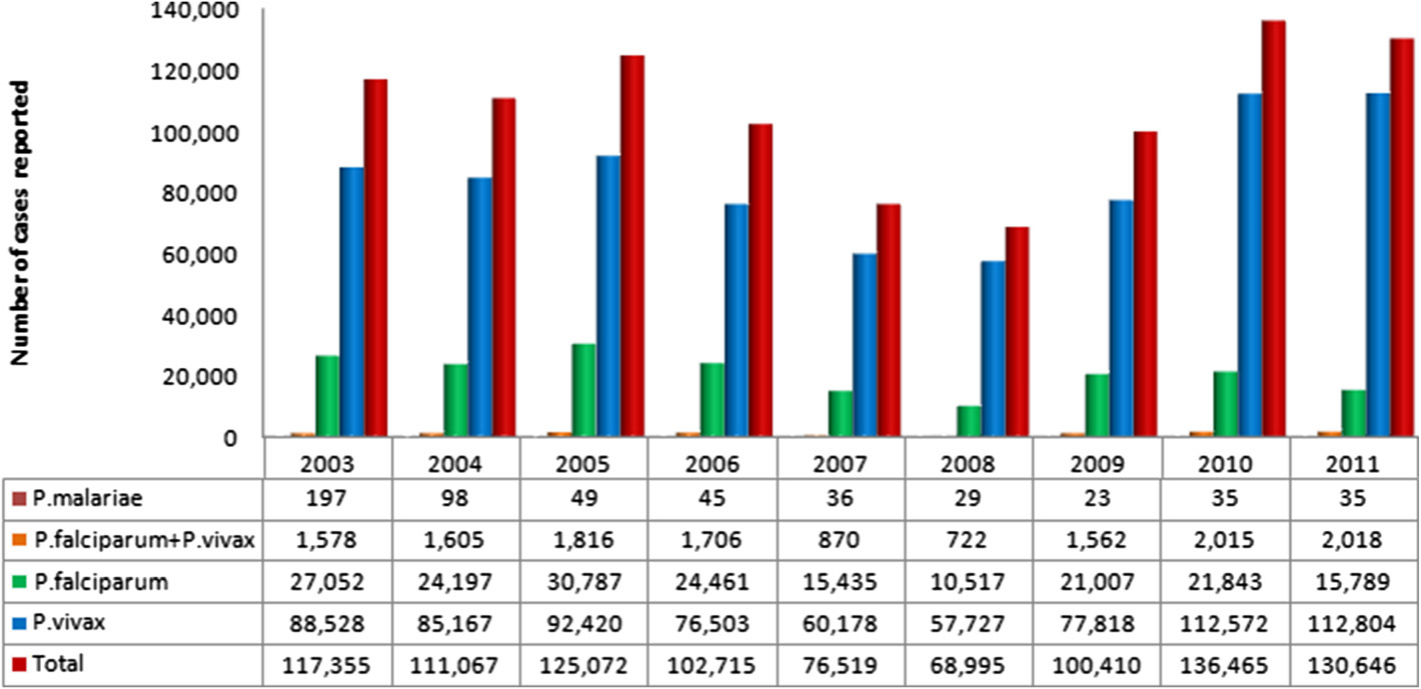
**Number of cases of malaria reported annually in the Brazilian state of Pará by *Plasmodium *** **species (P.) between 2003 and 2011.**

Between 2003 and 2011, there was a clear predominance of *P. vivax* in the cases of malaria reported in Pará (Figure 2). There was also a clear reduction in the number of cases reported for all *Plasmodium* species between 2006 and 2008. A more detailed analysis of the data for 2003 and 2011 by municipality and mesoregion, based on the risk of contracting malaria (API), indicated considerable contrasts between the different parts of the state. Based on this classification, the populations of 61 (42.66%) of the state’s 143 municipalities returned a moderate to high risk of contracting malaria in 2003. The south-western mesoregion was the worst affected, with half of its municipalities classified as high risk. In 2011, by contrast, 47 (32.87%) of the municipalities were classified as moderate (22.38%) or high risk (10.49%) for malaria, once again with the worst scenario being recorded in the south-western mesoregion, where 42.86% of the municipalities were classified as high risk.

In 2003, the highest rates of malaria were recorded in the Southwest, Southeast, and Marajó mesoregions, with APIs of 66.78, 29.97, and 28.14, respectively (Table 1). The scores indicate that the south-western mesoregion suffered the highest rates of infection throughout the study period, although the API did fall below 50 in 2008, but then rose again in subsequent years. Alarming increases in the APIs were recorded in the northeastern mesoregion (a 95.31% increase between 2003 and 2011) and the Marajó Archipelago (92.73%), whereas a considerable decline (78.83%) was recorded in the south-eastern mesoregion. The API for the state as a whole increased only 3.43% between 2003 and 2011, however.

**Table 1:** The API scores by mesoregion for the Brazilian state of Pará between 2003 and 2011

**Mesoregion**	**API**^**1 **^**score recorded in (year):**									**Variation**^**2 **^**(%)**
	**2003**	**2004**	**2005**	**2006**	**2007**	**2008**	**2009**	**2010**	**2011**	
Lower Amazon	9.44	9.63	10.53	10.97	11.60	9.04	8.99	6.38	8.41	-10.94
Belém Metropolitan Area	0.78	0.60	0.58	0.41	0.31	0.12	0.09	0.04	0.07	-90.52
Marajó Archipelago	28.14	17.97	43.67	34.36	21.62	25.50	54.35	72.91	54.24	92.73
Northeast	13.70	17.65	13.25	8.91	6.57	7.00	9.52	18.41	26.76	95.31
Southeast	29.97	25.25	24.37	16.70	10.32	8.38	9.93	11.18	6.35	-78.83
Southwest	66.78	76.58	85.99	79.17	59.80	42.98	51.16	71.53	72.67	8.82
Total	17.08	16.60	18.69	14.96	10.70	9.53	13.86	18.26	17.67	3.43

^1^API: Annual Parasite Index; ^2^2011 compared to 2003.

Based on the data from all 143 municipalities, the KMO index was 0.71, which indicated that the data were appropriate for the factorial analysis, and the result of the Bartlett test (*p* < 0.001) confirmed that the correlation matrix was significantly different from the identity matrix. Given this, the factorial analysis was applied for the extraction of the factors and the estimation of their scores (Table 2).

**Table 2:** Results of the factorial analysis

**Factor**	**Variable**	**KMO**	**Bartlett ( *p *)**	**% variance**	**Factor loading**	**Comm.**
1	Per capita income	0.71	< 0.001	47.05	0.91	0.83
	Literacy rate				0.59	0.69
	Public sanitation				0.74	0.55
	Refuse collection				0.79	0.72
2	Public water supply			26.63	0.94	0.89

Legend: Comm. - communality.

Two of the factors identified explain 73.68% of the total variance in the data. The selection of the variables for each of the factors is based on the factor loading, and in the present case, factor 1 has four loadings, while factor 2 has just one. The first factor, denominated the socio-economic index, includes the variables per capita income, literacy rate, public sanitation, and refuse collection. The variable that defines the second factor is the public water supply, and this was the denomination used for this factor (Table 2).

Following the identification of the factors, it was possible to calculate the factor scores. The sum of the scores for factors 1 and 2 was considered to be the Quality of Life Index (QLI), with scores being determined for each municipality, where the highest scores indicate the best quality of life. The classification of the indices by percentile permitted the division of the municipalities into groups, according to their quality of life scores, ranging from poor to good. The distribution of the classes among the different mesoregions was highly heterogeneous, indicating that this classification of the municipalities using these criteria was adequate. In particular, it was not that the percentage of households connected to a public sanitation is well below those with refuse collection and access to a public water supply.

In 2011, the QLI indicated that 99 (69.23%) of the 143 municipalities of the state of Pará had a poor or regular quality of life (Table 3), with the worst situation being found in the Northeast (92.11%), Southwest (85.71%) and Lower Amazon (64.29%). In stark contrast, all but two (81.82%) of the municipalities of the Metropolitan area of Belém were classified in the highest quality class.

**Table 3:** Number and percentage of municipalities in the different mesoregions of the Brazilian state of Pará according to their quality of life classification in 2011

**Mesoregion**	**Number (%) of municipalities by classification of the quality of life as:**			**Total**
	**Poor**	**Regular**	**Good**	
Lower Amazon	3 (21.43)	6 (42.86)	5 (35.71)	14
Belém Metropolitan Area	-	2 (18.18)	9 (81.82)	11
Marajó Archipelago	11 (40.74)	4 (14.81)	12 (44.45)	27
Northeast	16 (42.11)	19 (50.00)	3 (7.89)	38
Southeast	11 (28.21)	15 (38.46)	13 (33.33)	39
Southwest	9 (64.28)	3 (21.43)	2 (14.29)	14
Total	50 (34.96)	49 (34.27)	44 (30.77)	143

A correspondence analysis was used to verify the potential relationship between quality of life (QLI) and the risk of contracting malaria, based on the API. The initial *χ*^2^ analysis indicated that the two variables are associated significantly (*p* = 0.008), while the value of the β criterion (4.93) indicates that, in addition to the variables in themselves, their different classes are also associated. These parameters confirmed that the variables satisfied the conditions necessary for the application of the correspondence analysis.

The residuals of the probabilities derived from this analysis are considered to be significant when their values are above 70%. The results of the analysis indicate that there is a moderate to high risk of contracting malaria in municipalities with a low QLI – probabilities of 80.97% and 95.13%, respectively. By contrast, the risk of infection with malaria was low (79.24% probability) in municipalities classified as having good quality of life.

## Discussion

The results of the present study confirm that malaria is endemic to the Brazilian state of Pará, in particular in the poorer regions of the state. The disease thus constitutes a major public health problem for the Brazilian Amazon region, which was responsible for only 5.3% of the national GNP in 2010 [4], but accounts for 98% of the country’s cases of malaria. In 2011, 86% of the recorded cases were caused by *P. vivax* (Figure 2), a rate similar to that recorded in previous years [7].

In Pará, the variation in the number of reported cases between 2003 and 2011 indicates that the incidence of the disease continues to grow, with an increase of 3.43% in the API in 2011 in comparison with 2003 (Table 1). The environment does not only determine the distribution and incidence of the disease. The transmission of malaria appears to be favoured by conditions of poor sanitation conditions, which provide opportunities for host infection. Additionally individual characteristics, such as genetic predisposition, acquired immunity, age, family composition, occupation, education, religious beliefs, and culture are also important. The different species of parasite and vector found in the local environment should also be taken into consideration [7-9].

The potential effects of a number of geographic and environmental factors on the transmission rates of malaria are also well documented [10,11]. These include natural features, such as high temperatures, rainfall, and humidity, as well as anthropogenic modifications of the environment, such as deforestation and ongoing colonization, based on the establishment of settlements with inadequate living conditions, and the generally adverse conditions of rural life. All this factors contribute to the proliferation of malaria in the Amazon region.

Important divergences in results have been found in studies that have analysed the potential relationships between the incidence of malaria and socio-economic indices such as personal income, education, and public services, which appear to be related to the different approaches used to measure the incidence of malaria and poverty levels [12]. Given the practical difficulties of collecting reliable epidemiological and socio-economic data directly from the study areas, the approach adopted in the present study, based on the set of official statistics available for the 143 municipalities of the state of Pará, can be considered to be valid, due to the combination of ready access to reliable and standardized data and the low cost of the procedure. The results of the present study indicate that this approach is effective for the evaluation of the potential effects of socio-economic and environmental factors on the risk of infection with malaria.

Marked differences were found in this risk of infection in the different regions of the Brazilian state of Pará, considering the 143 municipalities included in the analysis. At least a third of these municipalities were characterized as moderate to high risk areas for the transmission of malaria, in particular the 15 (10.49%) that returned API scores of over 50 in 2011. The worst affected regions were the Marajó Archipelago and the Southwest, whereas the number of municipalities with high risk scores in the Southeast and Northeast declined considerably during the course of the study period.

In the specific case of the south-eastern mesoregion, the improvements in the infection rates observed during the study period may have been related to the evidence of economic growth in the region, which has resulted in improvements in the living conditions of the local population, with increasing investments in basic infrastructure, such as public water supplies and sanitation, the electric grid and paved streets. These advances have a positive effect on the prevention of the disease in the less economically privileged sectors of the society, and almost certainly contributed to the reduction in the numbers of reported cases and the incidence of malaria in the different municipalities [13].

However, the rural population is known to be more vulnerable to infection with malaria than urban residents. This is clearly shown in the results of the present study, given that the regions with the lowest indices of quality of life – Northeast, and Southwest – are characterized by lack of infrastructure, with much of the population living along rivers, creeks, and lakes, often in close proximity to swamps and marshes, and other areas prone to flooding, which represent ideal habitats for the proliferation of anopheline mosquitoes. This obviously leads to an increased incidence of malaria in these areas, and contributes to the considerably different transmission rates recorded in the different mesoregions of the state.

In addition, it is important to point out that malaria is endemic in the nine States of the Brazilian Amazon Region, where the number of cases has fluctuated over the years [7]. The analysis of the reasons for malaria transmission points to the social and economic development linked to an inadequate management of the environment, whose effects of the forest disturbances from selective logging, forest fires and road construction have been contributing to the highest malaria risk in some States of the Brazilian Amazon [14].

Comparing the incidence of malaria (API) at the start (2003) and end (2011) of the study period, it is clear that infection rates were highest in the Southwest, and increased 8.82% over the study period, while the rates in the Northeast and the Marajó Archipelago increased by more than 90%. In the Southeast, by contrast, the infection rate declined considerably, by 78% (Table 1). As seen in other developing countries where the incidence of malaria is high [15-18], the economically less privileged sectors of the society are the most affected. Populations with reduced economic conditions also have less opportunity for treatment [19], and are thus more vulnerable to the effects of the disease, which can worsen to a fatal condition, in some cases.

The present study indicates a systematic association between the QLI, based on a range of socio-economic variables, and the risk of contracting malaria (based on the API) in the municipalities of the Brazilian state of Pará. In the specific case of per capita income – an index of economic development – there was a clear negative relationship with the API, which decreased progressively with increasing income, as seen in the Belém metropolitan area and the southeast of the state. In other regions, such as the Marajó Archipelago and the Southeast, by contrast, the decline in incomes was accompanied by a marked increase in infection rates.

The categorization of the data for the state of Pará based on the quality of life index (QLI) resulted in the classification of 69.23% of its municipalities as either regular or poor (Table 3). The greatest concentrations of municipalities in these categories were identified in the Northeast, Southeast, and Marajó mesoregions, coinciding with the distribution of the municipalities with the highest risk of malaria, based on the API. The results of this study thus indicate a clear inverse association between the API and the QLI, that is, the municipalities having the lowest quality of life tended to have the highest risk of malaria. Similar findings have been reported in a number of previous studies [12,18,20].

However, many studies of this kind have produced inconclusive findings. A review of the literature found mixed results [19], with some studies demonstrating a significant association between poverty and malaria, whereas others found no such relationship. This contradiction has been explained by the methods used to classify malaria, given that, whereas studies based on personal reports of the disease or fever found no association, those based on parasitological data, as in the case of the present study, found a negative association between socio-economic status and the incidence of the disease [21].

Overall, the results of the present study indicate that, in addition to the local geographic and environmental factors that favour the transmission of the disease in the Amazon region, a set of secondary factors are also closely associated with the potential risk of infection with malaria (based on the API), in particular poverty. This scenario is defined by low income, inadequate public sanitation, water supply, and refuse collection, as well as low education levels. Many communities, in particular isolated, underprivileged rural settlements, present all these characteristics, reinforced by reduced access to preventive measures and treatment, and thus reinforcing the vulnerability of the poorest sectors of the population [22,23].

## Conclusion

The results of the present study revealed a close association between the quality of life of a community as well as the economic development to the general risk of contracting malaria. The municipalities of the State of Pará in Amazon region with the worst economic conditions and the less favourable development were identified as those that presented the highest risk of malaria transmission. It is important to note that malaria affects the productivity of the labour force, with negative consequences for economic growth, which restricts living conditions, favouring the dissemination of the disease in the population, which in turn suffer a permanent cycle of disease and poverty. Given this, investments are needed in basic infrastructure and education, measures that will contribute to the effective reduction of the incidence of malaria in the Brazilian Amazon region.

## Competing interests

The authors declare that they have no competing interests.

## Authors’ contributions

SGLJ initiated the writing of the manuscript. VMSP did the statistical analysis and interpreted the results. TCOC discussed the results and made the translation. EMLSR made critical revision of the manuscript start and end. All authors read and approved the final manuscript.
